# An Evaluation of a Web-Based Decision Aid for Treatment Planning of Small Kidney Tumors: Pilot Randomized Controlled Trial

**DOI:** 10.2196/41451

**Published:** 2022-09-02

**Authors:** Justin Fogarty, Mutita Siriruchatanon, Danil Makarov, Aisha Langford, Stella Kang

**Affiliations:** 1 Department of Radiology New York University Grossman School of Medicine New York, NY United States; 2 Department of Urology New York University Grossman School of Medicine New York, NY United States; 3 Department of Population Health New York University Grossman School of Medicine New York, NY United States

**Keywords:** small kidney mass, decision aid, renal tumor, randomized controlled trial, shared decision-making, decisional conflict

## Abstract

**Background:**

Surgery is the most common treatment for localized small kidney masses (SKMs) up to 4 cm, despite a lack of evidence for improved overall survival. Nonsurgical management options are gaining recognition, as evidence supports the indolence of most SKMs. Decision aids (DAs) have been shown to improve patient comprehension of the trade-offs of treatment options and overall decision quality, and may improve consideration of all major options according to individual health priorities and preferences.

**Objective:**

This pilot randomized controlled trial (RCT) primarily aims to evaluate the impact of a new web-based DA on treatment decisions for patients with SKM; that is, selection of surgical versus nonsurgical treatment options. Secondary objectives include an assessment of decision-making outcomes: decisional conflict, decision satisfaction, and an understanding of individual preferences for treatment that incorporate the trade-offs associated with surgical versus nonsurgical interventions.

**Methods:**

Three phases comprise the construction and evaluation of a new web-based DA on SKM treatment. In phase 1, this DA was developed in print format through a multidisciplinary design committee incorporating patient focus groups. Phase 2 was an observational study on patient knowledge and decision-making measures after randomization to receive the printed DA or institutional educational materials, which identified further educational needs applied to a web-based DA. Phase 3 will preliminarily evaluate the web-based DA: in a pilot RCT, 50 adults diagnosed with SKMs will receive the web-based DA or an existing web-based institutional website at urology clinics at a large academic medical center. The web-based DA applies risk communication and information about diagnosis and treatment options, elicits preferences regarding treatment options, and provides a set of options to consider with their doctor based on a decision-analytic model of benefits/harm analysis that accounts for comorbidity, age group, and tumor features. Questionnaires and treatment decision data will be gathered before and after viewing the educational material.

**Results:**

This phase will consist of a pilot RCT from August 2022 to January 2023 to establish feasibility and preliminarily evaluate decision outcomes. Previous study phases from 2018 to 2020 supported the feasibility of providing the printed DA in urology clinics before clinical consultation and demonstrated increased patient knowledge about the diagnosis and treatment options and greater likelihood of favoring nonsurgical treatment just before consultation. This study was funded by the National Cancer Institute. Recruitment will begin in August 2022.

**Conclusions:**

A web-based DA has been designed to address educational needs for patients making treatment decisions for SKM, accounting for comorbidities and treatment-related benefits and risks. Outcomes from the pilot trial will evaluate the potential of a web-based DA in personalizing treatment decisions and in helping patients weigh attributes of surgical versus nonsurgical treatment options for their SKMs.

**Trial Registration:**

ClinicalTrials.gov NCT05387863; https://clinicaltrials.gov/ct2/show/NCT05387863

**International Registered Report Identifier (IRRID):**

PRR1-10.2196/41451

## Introduction

Surgical resection remains the most common treatment for small kidney masses (SKMs; up to 4 cm in diameter, clinical stage T1a), though overall survival benefits have not been realized [1]. This troubling trend may be due to postsurgical worsening of kidney function and associated cardiovascular mortality in this generally older population with high rates of kidney disease, offsetting benefits of early tumor detection [2,3]. Although the majority of these SKMs are malignant, a small minority of tumors metastasize during a period of active surveillance, and approximately 20% are benign [1,4,5]. In fact, most kidney tumors are diagnosed as early-stage, incidental lesions on imaging tests performed for unrelated reasons [6,7].

Nonsurgical alternatives, such as percutaneous ablation and active surveillance with or without biopsy, may be considered to avoid surgeries in patients with indolent or benign tumors or in patients with risk factors for poor postsurgical outcomes. Therefore, we set out to determine the optimal management strategies for patients with SKMs and create evidence-based, patient-centered tools to communicate personalized harms and benefits of treatment options and promote shared decision-making. A decision-analytic model was developed to identify the key parameters, provide thresholds for variables affecting the decision (eg, test performance characteristics needed to improve outcomes), and assess the sensitivity of the decision to patient preferences [8]. We then interfaced the favored treatment options in accordance with patient and tumor characteristics with a decision aid (DA). DAs have shown benefit in these areas in treatment selection for prostate and breast cancer; while a DA has been published for kidney tumor management, consideration of specific features that can affect outcomes, such as chronic kidney disease (CKD) and tumor features, has had limited representation in such tools [9-11].

Several compelling reasons exist for exploring the development of a risk communication tool and its pilot testing among outpatients receiving kidney tumor treatment. First, there is a large amount of information regarding the diagnosis that patients may have difficulty understanding and remembering accurately in a single verbal discussion with a physician. Kidney tumors comprise a diverse group of different benign and malignant tumor types. Even among malignancies, there is a wide degree of variation in the potential to metastasize and cause cancer-related death [1,4,5]. Second, there are also additional tests (ie, imaging or biopsy) that can offer additional information about the potential of the patient’s tumor to progress, and these also require explanation that may not be effectively described by a nonradiologist.

Management options that serve as alternatives to initial surgical resection have gained recognition in clinical practice as reasonable alternatives to the current standard of surgery depending on the patient’s medical comorbidities and tumor features that affect the type of surgery recommended (partial or whole removal of the affected kidney). Partial nephrectomy is performed whenever possible to preserve some of the affected kidney but can still reduce kidney function, and baseline kidney disease is associated with worsened postoperative overall survival [12]. The guidelines of the American Urological Association do not describe specific criteria for offering these alternative forms of treatment [13,14]. Third, treatment-related consequences are important when selecting a treatment, and describing such complex medical consequences as CKD requires clear descriptions and ideally graphical representation. Fourth, this work aims to create a feasible model that, if effective, can be easily extended across diseases associated with possible personalized pathways of more testing options and delaying intervention when abnormalities are likely indolent, or initial selection of minimally invasive therapies. Thus, our goal is to assess the feasibility of use and preliminary effect of a web-based DA on treatment choice for patients with SKMs.

## Methods

### Study Design

The study is a single-blinded pilot randomized controlled trial (RCT; ClinicalTrials.gov NCT05387863) that will evaluate the preliminary effect of a web-based DA compared with the existing institutional website on treatment decisions and decision-making measures. The DA or existing institutional educational material and questionnaires will be administered through a web-based, integrated, Health Insurance Portability and Accountability Act–compliant platform. Participants will be randomized to either receive the newly developed web-based DA or the standard institutional material.

The DA will include evidence-based risk communication about the diagnosis and treatment options, interfaced results of a decision-analytic model for tailored benefits or harms assessment based on the oncologic and nononcologic risk factors for mortality (eg, patient characteristics, comorbidities, and tumor imaging features), and brief individual values clarification to incorporate into decision-making for SKMs. We will compare the routine counseling for treatment decisions against this DA. The treatment strategies represented in the tool will include 4 options: the current standard of care (initial surgery with partial or radical nephrectomy), and the nonsurgical options of percutaneous ablation, active surveillance with biopsy, and active surveillance without biopsy.

### Study Setting and Recruitment

**Figure 1:**
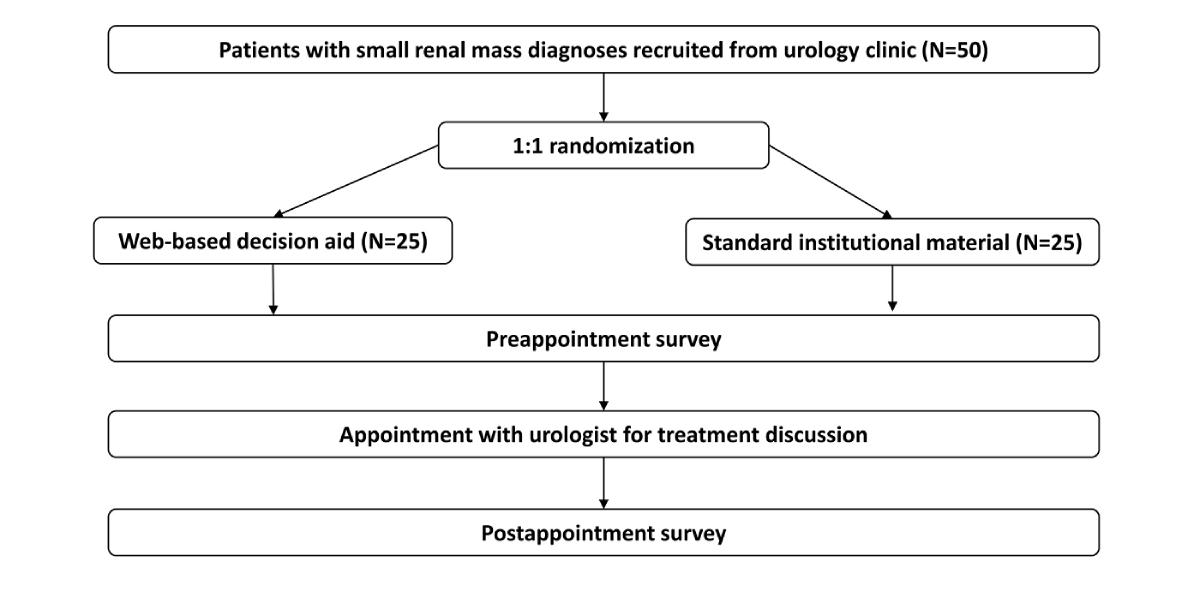
Patient randomization schema using 1:1 allocation to web-based decision aid or to standard institutional material.

This study will take place at 2 urology clinics within a large urban tertiary academic medical center in the northeastern United States. Patients will be identified via the clinic schedule in the electronic health record, where the reason for an upcoming first urologic consultation (ie, whether the patient has a newly diagnosed SKM) is specified. Study team members will then review the patient’s medical record to ensure the SKM meets the size criteria (up to 4 cm), there is no regional or distant metastatic disease on imaging, and that no other exclusion criteria are present. Once identified, patients will be recruited via the telephone or email prior to their standard of care consultation with their urologist. They will have already received a diagnosis by routine communication with their care team. Patients will be aged 18 years or older and diagnosed with a localized renal tumor (clinical stage T1a) for which they have not yet received treatment. Other exclusion criteria are stage IV cancer of any type, vulnerable subjects, inability to understand English, and inability to provide informed consent. When a patient agrees to participate, a member of the study team will meet them in the urology clinic on our medical center’s campus prior to their appointment to begin the study visit. The web-based DA will be administered to 25 patients, and the existing institutional website will be shown to 25 patients who provide informed consent. Patients will be randomized using a 1:1 allocation ratio (DA comprises standard institutional materials; see Figure 1) with a random sequence generated by a study team member to assign patients and with blinding of the investigators.

### The Web-Based Decision Aid (Intervention)

The web-based DA was created by an interdisciplinary committee beginning in 2020, including a diagnostic and interventional radiologist, urologists, internist, and experimental psychologists who agreed on essential design and content components. Through an iterative process, team members designed and tested the DA in small groups of SKM patients to continuously improve on its contents. In its nascent form, the DA was a printed booklet designed in accordance with the International Patient Decision Aid Standards [15]. Through focus groups and semistructured interviews with patients with SKM and 3 physicians, thematic analysis was also conducted to improve this prototype DA until the printed version was finalized. Upon identifying additional educational needs through knowledge items, the web version incorporated more graphics for explaining CKD, the impact of CKD on overall health, and R.E.N.A.L. Nephrometry scores—a standardized measure of anatomic complexity [16].

The web-based DA tool delivers information regarding SKMs, treatment options, and preparation for shared decisions to improve patient knowledge and provide tailored treatment options based on patient information. With the additional web-based graphics and values clarification, the tool may further improve upon initial findings that the booklet form of the DA resulted in more accurate understanding of risks associated with SKMs and each treatment option, and greater preference for nonsurgical initial approaches as compared to patients who received existing institutional educational materials [17].

**Figure 2:**
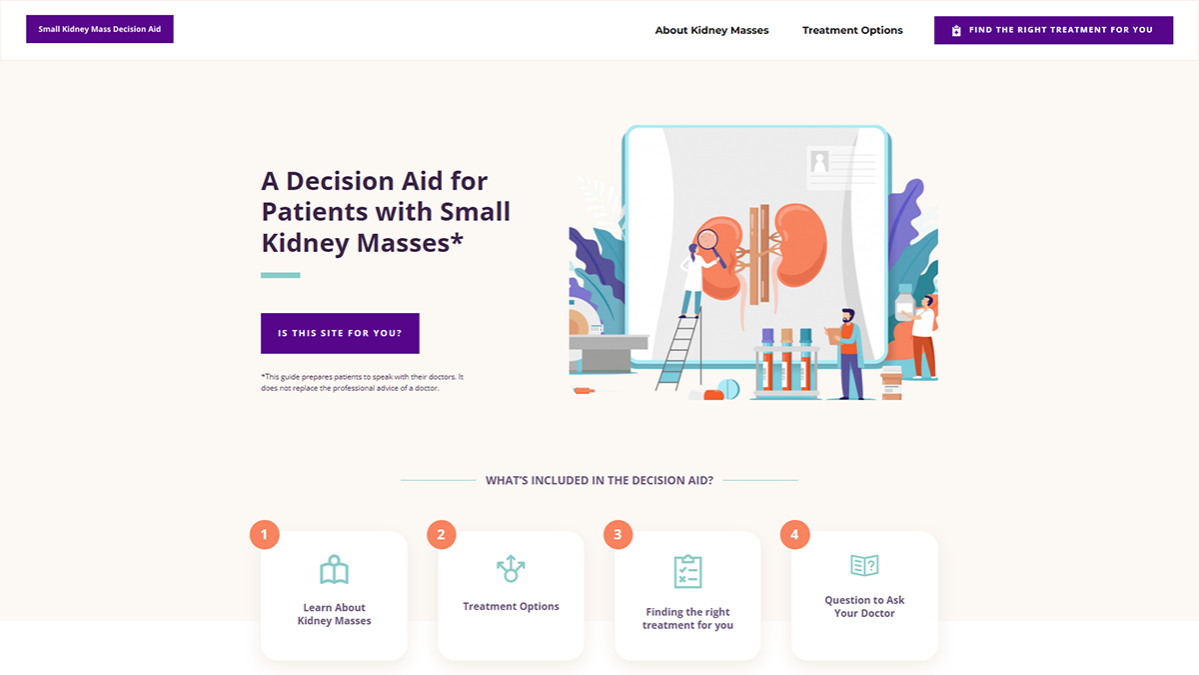
The landing page for the DA website contains a brief introduction of the contents patients will see while using the DA.

**Figure 3:**
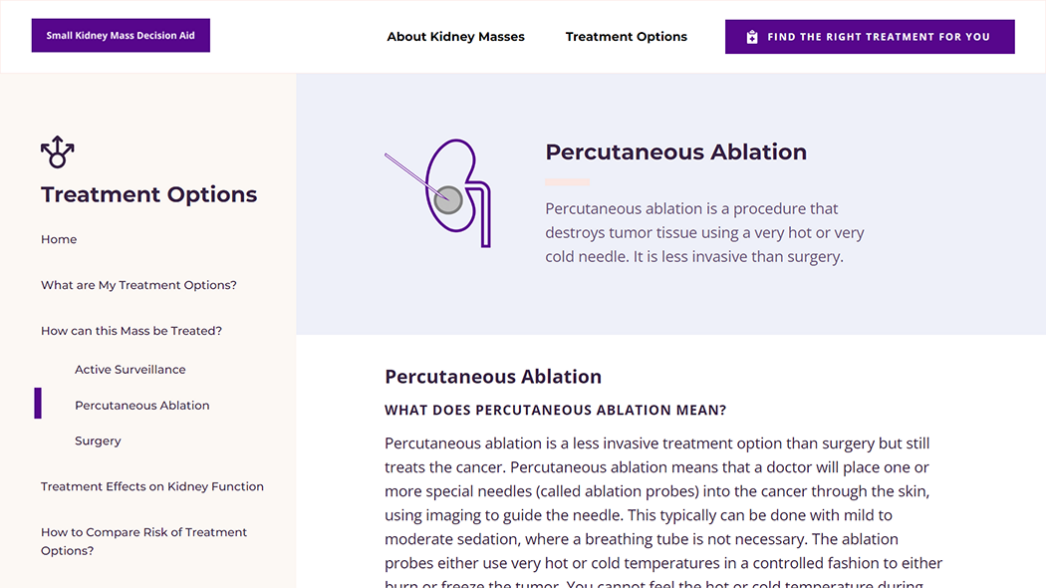
Screenshot of a page of the web-based decision aid providing information on one of the treatment options—percutaneous ablation.

The DA states the advantages and disadvantages of each treatment option and is designed to prepare for informed engagement in a treatment discussion with the urologists at the first clinical consultation after diagnosis of the mass. The DA tool is a set of web pages with 3 main sections (*About Small Kidney Masses*, *Treatment Options*, and *Find the Right Treatment for You*) and content covering cancer staging information, normal kidney function, chronic kidney disease, and a tool to provide tailored treatment recommendations (Figures 2 and 3). The first topic captures an overview of the kidney and its function, CKD and its progression, SKMs, the types of tumors (benign vs malignant) and how each type could progress, and the risk factors leading to kidney cancer. The second topic focuses on SKM management, with treatment options ranging from active surveillance to minimally invasive procedures (percutaneous thermal ablation) to surgery. For each option, we provide an overview of how the treatment is performed including its benefits and potential effects on kidney function and key factors that could increase the risk of harm from treatment. All information is provided with images for clarification as needed, and educational content is written in consideration of literacy at an 8th grade reading level.

**Figure 4:**
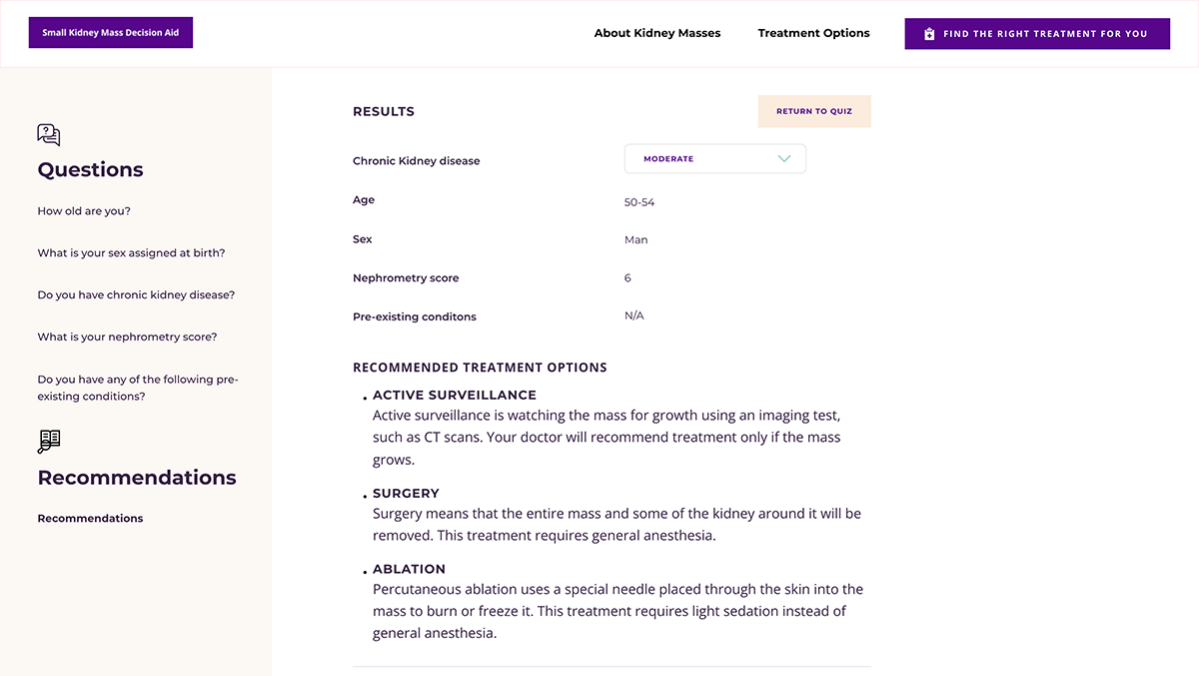
Screenshot of treatments to consider with providers after patients indicate responses regarding their age, health, and tumor characteristics. CT: computed tomography.

The third section of the web-based DA includes 5 questions on age, sex, known diagnosis of CKD and CKD stage, R.E.N.A.L. Nephrometry score, and a list of comorbidities for which patients indicate presence or absence. Any missing responses can result in a default answer that applies a normal baseline value (or minimum Nephrometry score) with instruction to discuss these factors and treatment options with their doctor. To facilitate shared decision making, patients are asked to rate the importance (scale of 1-5, with 5 being very important), of attributes of the treatment choices (possible advantages and disadvantages of each treatment option) and then to indicate the preferred treatment option at that time. These features will allow evaluation of the most common and important preferences in treatment decisions. Finally, the ratings for treatment attributes are presented along with the set of treatment choices that should likely be considered with the doctor, based on presence or absence of risk factors for poor surgical outcomes (Figure 4).

### Procedure

Data time points comprise one visit for each participant. The study visit, including the patients’ appointments with their urologists, will last approximately 2 hours. Patients will arrive for their regularly scheduled appointments 50 minutes early. Patients who agree to participate will go through the informed consent process with a member of the study team and will subsequently be randomized to receive the DA or the standard, web-based institutional pamphlet (Figure 1). Prior to their consultation with their urologist, they will answer brief surveys for demographics and literacy or numeracy [18-20]. They will then review the web-based DA or standard institutional material provided by the research team. The study team member will be available only for technical questions while reviewing the materials. Patients will then answer surveys about the material provided, their treatment preferences, and knowledge items (Multimedia Appendix 1) about SKMs’ treatment options.

After reviewing the educational materials and answering the first sets of surveys, patients will attend their appointment with their urologist. Following their consultation, the patients will answer surveys about the visit and the decision-making process and will reindicate their desired treatment at that time.

In the following 3-6 months, a study team member will check patient medical records to assess what management option the patient undertook for the first 3 months after the initial consultation, kidney function at the clinically indicated follow-up visits (typically at least one blood test within 6 months regardless of the treatment), routine blood tests, and reports of tumor stage progression.

The questionnaires administered throughout the study, the web-based DA, and standard institutional website will be administered on electronic tablets while a member of the research team is present to answer technical questions about questionnaire functionality and website navigation problems. We will use the institutional Research Electronic Data Capture system to create and administer surveys [21,22]. An advantage of this software is its ability to create unique links leading to the DA website or the web-based institutional materials, creating a seamless integration of survey responses and DA use and responses. Patients will receive US $50 gift cards as compensation for time spent in review of the study materials.

### Measures

Prior to reviewing the educational materials, participants will answer questionnaires to collect demographic information (sex, race and ethnicity, and educational attainment), self-described literacy abilities [18], numeracy abilities [19,20], and comfort with using different types of technology. A primary outcome for this pilot study is feasibility of viewing the web DA prior to the appointment, and thus the percentage of patients viewing most or all of the tool will be assessed. Participants will answer questions regarding SKM knowledge after presentation of the educational materials to assess the educational benefit of the DA versus the standard institutional materials. Participants will also indicate their preferred treatment. Just after the patient finishes the urologic consultation, an additional survey will be administered. In this postvisit survey, the questions will include the Decisional Conflict Scale, which consists of 16 prompts with a 5-point Likert scale ranging from 1=“strongly disagree” to 5=“strongly agree” to assess uncertainty in the decision, decision efficacy, and contributors to feelings of uncertainty [23,24]. A second survey assessing the shared decision-making (SDM) process scores will be administered to measure patients’ ability to share during decision-making while considering each treatment’s pros and cons [25]. In the postvisit survey, we ask participants again to indicate their preferred treatment. The measures used for our study analysis are provided in Table 1.

Renal function will also be recorded as an exploratory measure, as well as other major comorbidities represented in the Charlson comorbidity score [26], through a review of medical records for up to 6 months following a participant’s completion of the study.

**Table 1:** Decision-making measures and their corresponding descriptions.

Measure		Description
3-item Subjective Numeracy Scale		Individual numeracy skill based on a summation of self-reported ratings of 3 items, including individual comfort to fractions, percentage, and numerical information, where the rating scale ranges from 1=“not good at all” to 6=“extremely good.”
**Decisional Conflict Scale (DCS)**		Difficulty in decision-making based on a questionnaire consisting of 16 items with responses rated on a 5-point Likert scale ranging from 1=“strongly disagree” to 5=“strongly agree.” To calculate DCS scores, we will convert the responses of 1 to 5, 2 to 4, and from 5 to 1 such that the DCS range is from 0=“no decisional conflict” to 100=“extremely high decisional conflict.” DCS has 5 subscores: uncertainty, informed, values clarity, support, and effective decision subscores.
	Uncertainty subscore	Measure of uncertainty or confidence in patient decision-making based on 3 items (items 10-12) from a 16-item questionnaire. The subscores are calculated by (1) summing, (2) dividing by 3, (3) deducting by 1, and (4) multiplying by 25.
	Informed subscore	Measure of how well-informed patients are regarding treatment options based on 3 items (items 1-3) from a 16-item questionnaire. The subscores are calculated by (1) summing, (2) dividing by 3, (3) deducting by 1, and (4) multiplying by 25.
	Values Clarity subscore	Measure of patient clarity in their preference regarding treatment benefits and harms based on 3 items (items 4-6) from a 16-item questionnaire. The subscores are calculated by (1) summing, (2) dividing by 3, (3) deducting by 1, and (4) multiplying by 25.
	Support subscore	Measure of how much support or advice patients receive from others influence their decision based on 3 items (items 7-9) from a 16-item questionnaire. The subscores are calculated by (1) summing, (2) dividing by 3, (3) deducting by 1, and (4) multiplying by 25.
	Effective Decision subscore	Measure of how effective or satisfied patients feel about their decision based on 4 items (items 13-16) from a 16-item questionnaire. The subscore is calculated by (1) summing, (2) dividing by 4, (3) deducting by 1, and (4) multiplying by 25.
	Total scores	The total score is calculated by (1) summing, (2) dividing by 16, (3) deducting by 1, and (4) multiplying by 25.
**Shared Decision-Making (SDM) Process scores**		The degree of shared decision-making occurring during a treatment discussion between a patient and a clinician. SDM covers options, pros, cons, and preferences. The total score ranges from 0 to 4, where higher values represent a greater degree of shared decision-making.
	Options	Measure of discussion on each of the available treatment options based on yes/no responses. To calculate the score, we will (1) convert “yes” to 1 and “no” to 0 for each question and (2) calculate the average of all relevant questions.
	Pros	Measure of discussion on reasons patients should receive each of the treatment options based on the following responses: “a lot,” “some,” and “a little.” To calculate the score, we will (1) convert “a lot,” “some,” and “a little” to 1, 0.5, and 0, respectively, for each question and (2) calculate the average of all relevant questions.
	Cons	Measure of discussion on reasons patients should not receive each of the treatment options based on the following responses: “a lot,” “some,” and “a little.” To calculate the score, we will (1) convert “a lot,” “some,” and “a little” to 1, 0.5, and 0, respectively, for each question and (2) calculate the average of all relevant questions.
	Preferences	Measure of discussion on the selection of preferred treatment based on a yes/no response. To calculate the score, we will convert “yes” to 1 and “no” to 0.
	Total scores	Total score is a summation of options, pros, cons, and preferences.

### Sample Characteristics

We expect to enroll a total of 50 patients in this phase for the pilot trial. We expect that more adult men than women (approximately 60% men vs 40% women) will participate in this study based on the biologically higher incidences of kidney tumors in men. Based on previous research at our institution, we expect our sample to be approximately 25% Hispanic or Latino and approximately 25% Black or African American.

### Data Analysis

Demographics, literacy and numeracy, technology use, and both pre- and postconsultation treatment preferences will be compared between the DA and institutional educational material groups using the Mann-Whitney test for continuous variables. We will also use a Fisher exact test for proportions (R version 4.0.5 [27]) with the mid-p adjustment for a 2 × 2 contingency table (exact2x2 package [28]) and with the ordinary *P* value for an r × 2 contingency table where r>2 [27]. Missing responses will be excluded from the analysis. All statistical tests will be conducted at a 2-sided 5% comparison-wise significance level without adjustments for the number of comparisons.

### Ethics Approval

The initial phases of DA development consisting of focus groups, semistructured interviews, and initial usability testing of the printed DA with patients with SKM), as well as the current phase pilot RCT protocol were approved by the New York University Grossman School of Medicine’s Institutional Review Board (s16-010008 and s21-01670).

## Results

The pilot RCT using the web-based DA was funded by the National Cancer Institute on August 10, 2021. After testing the web tool, enrollment will begin on August 29, 2022. We estimate recruitment and data collection to be completed by December 31, 2022, with analysis completed by January 30, 2023.

## Discussion

### Principal Findings

The study aims to evaluate the feasibility of administering of a web-based DA in the clinic and assessing its effects on decision-making compared with an existing institutional website explaining treatment outcomes of patients with SKMs. We anticipate that the web-based DA will be a feasible intervention to enhance patient knowledge of SKMs, with >80% of participants viewing all of the tool and DA recipients having higher knowledge scores. Early-stage data will be collected on surgical or nonsurgical treatment selection, with the expected outcome of fewer DA recipients choosing surgery as an initial treatment option compared to recipients of the standard institutional material, though the pilot will be underpowered to detect a difference. We will conduct comparative evaluation of the knowledge of patients, decision satisfaction, decisional conflict, and shared decision-making as secondary measures, with expected increases in knowledge, decision satisfaction, and shared decision-making and decreases in decisional conflict among DA recipients.

### Strengths and Limitations

This study is the first example of a web-based DA for patients with SKMs that incorporates personalized risk-based information to guide treatment decisions. The rigorous approach to evaluating this intervention will pave the way for larger studies that aim to evaluate such web-based tools for patient viewing prior to a discussion with their providers about choosing a management approach. For incidental nodules in particular, accurate understanding of risk associated with the lesion itself (ie, metastasis and cancer-specific death), even if representing cancer, and the risks and benefits of treatment options are key to making decisions that are congruent with the degree of mortality risk. The DA was thus designed to allow patients with SKMs (nearly all incidentally detected) to understand their condition in an interactive, concise manner that facilitates better understanding of their options prior to their discussion with a urologist.

Initial usability testing in phase 2 informed an estimated review time of the given materials in under 30 minutes [17]. Patients who received the DA were more likely to self-report reviewing the DA completely than those who received the standard pamphlet [17], supporting the likelihood of viewing the web version of the tool, indicating that the DA is easily accessible to most populations and is not expected to be burdensome.

There are limitations to our proposed pilot trial, including the relatively small sample size for this initial evaluation of the web-based DA. The pilot RCT will be underpowered for detecting anticipated differences in surgical versus nonsurgical treatment selection with the use of a DA. There are elements of limitations inherent to more pragmatic trial designs, such as the ability of patients to access the tool on the internet with minimal usage direction by clinical staff instead of a highly controlled teaching session, self-report of the amount of content viewed in the DA, and minimal preparatory information or training of patients before using the tool. These elements were allowed for real-world evaluation of such decision support.

Several prior RCTs have been published on the role of DAs in prostate cancer treatment selection [9,11,29,30]. These trials have shown a broader range of treatment selected (ie, radiation therapy rather than prostatectomy) and increased decisional quality for patients. Similar to low-risk prostate cancer, treatment options for SKMs are associated with small differences in cancer progression between surgical treatment and nonsurgical therapy; thus, the trade-offs of choices warrant a DA.

### Dissemination Plan and Future Directions

Results will be disseminated through peer-reviewed publications and conference presentations. Results from this pilot study will also be used to further inform the design of a larger multisite RCT for use in urologic clinics. The pilot randomized testing may lead to further revisions for the web-based DA, and ultimately a future trial would evaluate the differences in treatment choice (surgical vs nonsurgical treatment selection) and clinical outcomes.

### Conclusions

While previous studies reported development of a DA for patients with SKMs, this study will address a gap in the existing literature. The newly developed web-based DA not only provides personalized, evidence-based risk communication, but also factors in tumor imaging features and potential comorbidities of surgical treatments as influencing factors in assessing patients’ treatment preferences, which may increase patients' satisfaction with their treatment decisions. Eventually, a DA for patients with SKMs may show similar impact on initial surgical treatment as prior trials on DAs for prostate cancer, with accompanying increases in SDM and decisional quality. If the web-based DA is demonstrably effective in increasing patient knowledge about their treatment options and in reducing decisional conflict, it could be considered an adjunct more broadly for patients with SKMs.

## Appendix

Multimedia Appendix 1 Original knowledge items for patients to answer after viewing the web-based decision tool.

Multimedia Appendix 2 CONSORT-eHEALTH checklist (V 1.6.1).

## Abbreviations

CKD: chronic kidney disease
DA: decision aid
RCT: randomized controlled trial
SDM: shared decision-making
SKM: small kidney mass

## Data Availability

The data sets generated from this study will be available upon study completion from the corresponding author on reasonable request.
