# Supplementary material for: An Evaluation of a Web-Based Decision Aid for Treatment Planning of Small Kidney Tumors: Pilot Randomized Controlled Trial
Source: JMIR Res Protoc. 2022 Sep 2;11(9):e41451. doi: 10.2196/41451 (PMC9482069; doi:10.2196/41451)
Supplement: Multimedia Appendix 1 [file resprot_v11i9e41451_app1.pdf]

Tool for Assessing Patient Knowledge about SKMs

1. Do most small kidney masses tend to grow rapidly or quickly?
    a. Rapidly
    b. Slowly
    c. Don’t know

2. For small kidney masses, how does having a mass near the center of the kidney affect the kind of treatment recommended?
    a. Worse chance of successful treatment with removal of the entire kidney
    b. Equal chance of successful treatment with any option
    c. Better chance of successful treatment with removal of the entire kidney
    d. Don’t know

3. In patients with kidney masses, does a larger size mean that they will have a worse, equal, or better chance that the treatment would be successful?
    a. Worse chance
    b. Equal chance
    c. Better chance
    d. Don’t know

4. Without treatment, about how many patients with small kidney masses will eventually die of kidney cancer?
    a. Most will die of kidney cancer
    b. Half would die of kidney cancer
    c. Most will die of some other cause
    d. Don’t know

5. For most patients with an early stage kidney cancer, how much would waiting 6-12 months to make a treatment decision affect their chances of survival?
    a. A lot
    b. Somewhat
    c. A little or not at all
    d. Don’t know

6. Which of the following are good reasons for a patient with a small kidney mass to consider NOT having treatment right away?
    a. To avoid or delay the possible side effects of these treatments
    b. To make sure these treatments are really needed
    c. Both to avoid or delay the possible side effects of these treatments and to make sure these treatments are really needed
    d. Don’t know

7. Of the following treatments for small kidney masses, which can cause problems with how well the kidney functions?

<u>Surgery:</u>

   a. Yes
   b. No
   c. Don't know

<u>Ablation:</u>

   a. Yes
   b. No
   c. Don't know

<u>No Active Treatment Right Away:</u>

   a. Yes
   b. No
   c. Don't know

8. How does chronic kidney disease affect how long a patient will live, also called life expectancy?
   a. Shorter life expectancy
   b. Equal life expectancy
   c. Better life expectancy
   d. Don't know
